# Analysis of the typing of adenovirus and its clinical characteristics in children with acute respiratory tract infection

**DOI:** 10.1186/s12887-023-03840-6

**Published:** 2023-01-16

**Authors:** Li Wang, Xiaoting Hu, Zhenzhen Huang, Yangjie Zhang, Xiaoyuan Zhao, Xiaohua Liu, Hua Mao, Huixiang Hao, Wanli Xue

**Affiliations:** 1 Nosocomial Infection Management Office, The Second Affiliated Hospital of Xi’an Jiaotong University, Xi’an, Shaanxi China; 2 Department of Pediatrics, The Second Affiliated Hospital of Xi’an Jiaotong University, Xi’an, Shaanxi China; 3 Department of Nutrition and Food Safety Research, School of Public Health, Xi’an Jiaotong University Health Science Center, No.76 Yanta West Road, Yanta District, Xi’an, 710061 Shaanxi China

**Keywords:** Acute respiratory tract infection, adenovirus, Typology, Clinical features

## Abstract

**Background:**

The purpose of this study was to investigate the typing of adenovirus (AdV) infection in children hospitalized with acute respiratory tract infection (ARTI) and its clinical characteristics.

**Methods:**

Samples from 7832 hospitalized children with ARTIs from January 2021 to June 2022 were tested by multiplex PCR for AdV. AdV hex neighborhood genes were amplified and sequenced for typing by nested PCR.

**Results:**

Three hundred twenty-eight cases were positive for AdV with rate of 4.48% (328/7832). No statistical difference in the rate of AdV detection was observed in different ages (*P* > 0.05). Among the 328 cases, 305 cases underwent amplification and sequence determination of AdV five-neighborhood, six-neighborhood and fibronectin genes. Only 237 cases were sequenced successfully for all 3 genetic fragments. The typing results of 231 cases with 3 genes were consistent, with 49.78% (115/231) of type 3, 41.56% (96/231) of type 7 and 8.66% (20/231) of other types identified. The main clinical symptoms in 231 children hospitalized with ARTI who were AdV positive were cough, sputum not easily coughable, Wheezing or shortness of breath and fever. Clinical diagnoses of 231 cases included: acute bronchitis 3.03% (7/231), capillary bronchitis 16.45% (38/231), pneumonia (mild/severe) 76.62% (177/231) (68.40% (158/231) in mild and 8.23% (19/231) in severe cases), bronchial asthma combined with pulmonary infection 3.46% (8/231). Higher percentage of shortness of breath, multilobar infiltration, and pleural effusion were found in type 7. Calcitoninogen in type 7 were significantly higher than those of type 3 and other types, and the white blood cell count was lower than those of type 3 and other types, and the difference was statistically significant (*P* < 0.05).

**Conclusion:**

AdV type 3 and 7 were frequently found in hospitalized children with acute lower respiratory tract involvement. AdV type 7 seems to be associated with more severe outcome.

## Introduction

According to relevant statistics, ARTI account for the highest morbidity and mortality among children under 5 years of age, with 120–156 million illnesses per year, of which about 920,000 die, and about 95% of the deaths are in low- and middle-income countries [1, 2]. Thus, ARTI are one of the leading causes of morbidity and mortality in children worldwide, and respiratory diseases caused by AdV infections account for 5–7% [3]. AdV are capable of causing a range of diseases, including ARTI, acute gastroenteritis, conjunctivitis, hemorrhagic cystitis, and meningoencephalitis [4, 5]. Previous evidence points out that AdV serotypes 1, 2, 3, 4, 5, 6, 7, 11, 14, and 21 are mainly associated with respiratory diseases, with AdV types 3, 4, 7, and 14 being the main types causing ARTI in children [6]. In recent years, the newly emerged type 14PI and recombinant type 55 and AdV types 3, 4, 7 and 14 often cause severe lower respiratory tract infections and are the main pneumonia agents of fatal incendiary infections in infants and children. Differences in viruses lead to different diseases, while small changes in the genes of the same virus can have an important impact on the viral pathogenicity [7]. Among the same virus, there are differences in clinical symptoms, disease severity and prognosis triggered by different types [8]. Therefore, investigation of the typing and clinical characteristics of AdV infection in children is a hot topic of clinical research, however, there are few clinical studies on this topic. Based on this, this study will investigate the typology and clinical characteristics of AdV infection in children.

## Materials and methods

### Clinical data

Retrospective analysis of 7832 respiratory specimens sent from children admitted to our hospital with ARTI from January 2021 to June 2022 were tested for AdV positivity. The study was in accordance with the relevant requirements of the World Medical Association Declaration of Helsinki [9]. Inclusion criteria: all children met the diagnostic criteria for ARTI in Zhu Fu-Tang Practical Pediatrics [10]; all children hospitalized with ARTI in the Department of Respiratory Medicine and the Department of Critical Care Medicine of our hospital; respiratory specimens collected on the day of admission or on day 2 were tested positive for AdV antigen by direct immunofluorescence assay (DFA, Diagnostic Hybrids, USA) or AdV nucleic acid positivity by respiratory pathogen detection kit (RPP, Luminex, USA); informed consent signed by the guardians of the children. Exclusion criteria: guardians who did not want to participate in this study; children with severe cardiopulmonary disease, immunosuppression, or immune ischemic disease.

The study protocol was approved by the Ethics Committee of Xi’an Jiaotong University Health Science Center. Informed consent was obtained from all the legal representatives or the guardian of the children participants involved in the study.

### Processing of specimens

Specimens such as nasopharyngeal swabs, nasopharyngeal secretions, sputum and alveolar lavage fluid were collected from the children, 2 ml of Hank’s fluid was added to the blown and mixed, and centrifuged at 1200 g for 10 min. The supernatant was partially used for viral nucleic acid extraction for RPP detection, and the remainder was stored at − 80 °C; the precipitate (mainly exfoliated respiratory epithelial cells) was resuspended and spotted onto slides for DFA detection. RPP and DFA were performed according to the kit instructions.

### Nucleic acid extraction and AdV typing

Specimens tested positive for AdV by DFA and RPP were selected, 140 μl of specimen supernatant was taken, nucleic acid extraction kit from QIAGEN, total nucleic acid was extracted according to the instructions, and finally dissolved in 60 μl of buffer and stored at − 20 °C. PCR was performed to amplify each of the major coat protein genes of AdV five-neighbor, six-neighbor and fibronectin. The PCR products were sequenced using the ABI13730xI DNA analyzer (Beijing sinogenomax Co.)

### Phylogenetic analysis

After the sequencing results were returned, Chromas Lite 2.22 software was applied to determine the correct reading of the chromatogram of the sequencing results, and NCBI BLAST was used to determine whether the amplification products were AdV, and the preliminary determination of the type; Editseq and Seqman in DNA Star were used to splice and edit the sequences. Clustal W in MECA X software was used to compare the obtained sequences with the reference strains of the identified type in GenBank, and the neighbor-joining method was used to construct a phylogenetic tree based on gene distances.

### Collection of clinical data of patients

Including the age, gender, clinical diagnosis, admission time, discharge time, length of stay, discharge department, and imaging data of the children.

### Statistics

SPSS 23.0 software was applied to analyze the data. Continuous variables with normal distribution were described by mean ± sd, and independent samples t-test was used for comparison between groups; continuous variables with non-normal distribution were described by M(Q1,Q3), and U-test was used for comparison between groups; count data were described by cases (%), and chi-square χ2 test was used. Differences were considered statistically significant at *P* < 0.05.

## Results

### AdV positivity rate in children hospitalized with ARTI

Among the respiratory specimens sent from children hospitalized with ARTI, 328 cases were positive for AdV, with a positive detection rate of 4.48% (328/7832). The detection rate was 4.81% (194/4032) for boys and 3.53% (134/3800) for girls, with a positive detection rate of 2.97% (38/1281) for AdV ≤6 months of age, 4.86% (39/802) for AdV > 6 months of age to 1 year of age, 6.67% (83/ 1245), 6.38% for > 2–5 years (131/2054), and 2.16% for > 5 years (53/2450), with no statistically significant difference (*P* > 0.05).

### AdV genotyping

Among the 328 AdV-positive respiratory samples, 23 had insufficient volume for testing and the remaining 305 underwent amplification and sequencing of AdV genotypes. The 3 gene fragments of AdV were successfully sequenced in 237 cases. The results of phylogenetic tree typing showed that the typing results of 3 gene fragments in 6 cases were inconsistent and could not be determined. The genotyping results of 3 gene fragments in the remaining 231 cases were consistent., among which 49.78% (115/231) of type 3 AdV, 41.56% (96/231) of type 7, and 8.66% (20/231) of other types were identified.

### Main clinical symptoms of AdV positivity in children hospitalized with ARTI

The main clinical symptom of AdV positivity in 231 children hospitalized with ARTI was cough 96.54% (223/231), followed by having sputum that could not be easily coughed up 71.43% (165/231), then shortness of breath 74.03% (171/231), fever 47.19% (109/231), in addition to a few cases of nasal congestion 14.72% (34 /231), runny nose 15.15% (35/231), vomiting 12.99% (30/231), diarrhea 9.52% (22/231), convulsions 6.06% (14/231), and rash 2.60% (6/231).

Type 7 wheezing or shortness of breath proportion was significantly higher than type 3 and other types, and the white blood cell count was lower than type 3 and other types, and the difference was statistically significant (*P* < 0.05); with no difference when comparing gender, age, peak body temperature, cough, having sputum not easily coughing up, fever, nasal congestion, runny nose, vomiting, diarrhea, convulsions, and rash of type 7 and other types in the 3 groups, see Table 1.

**Table 1:** Demographic and clinical data of the hospitalized children with acute respiratory tract infection according to the Adenovirus types

Indicators	Type 3 (*n* = 115)	Type 7 (*n* = 96)	Other Type (*n* = 20)	χ^2^/t	*P*
Gender (male/female, n)	72(62.61)/43(37.39)	60(62.50)/36(37.50)	12(60.00)/8(40.00)	0.063	0.969
Age (years)	3.01 ± 0.37	3.09 ± 0.41	3.03 ± 0.43	0.458	0.633
Peak body temperature (°C)	39.37 ± 0.78	39.42 ± 0.83	39.47 ± 0.87	0.183	0.833
Cough	110(95.65)	94(97.92)	19(95.00)	0.957	0.620
Sputum not easily coughed up	83(72.17)	69(71.88)	13(65.00)	0.477	0.788
Shortness of breath or shortness of breath	12(10.43)^#*^	25(26.04)	2(10.00)	0.874	0.013
Fever	54(46.96)	45(46.88)	10(50.00)	0.069	0.966
Nasal congestion	16(13.91)	13(13.54)	5(25.00)	1.848	0.397
Runny nose	17(14.78)	14(14.58)	4(20.00)	0.402	0.818
Vomiting	15(13.04)	12(12.50)	3(15.00)	0.092	0.955
Diarrhea	11(9.57)	9(9.38)	2(10.00)	0.008	0.996
convulsions	7(6.09)	6(6.25)	1(5.00)	0.046	0.977
Rash	3(2.61)	2(2.08)	1(5.00)	0.557	0.757

^#^*P* < 0.05 compared with type 7

^*^P < 0.05 compared with other types

### Clinical diagnosis of AdV positivity in children hospitalized with s

The 231 AdV-positive clinical diagnoses in children hospitalized with ARTI included: acute bronchitis 3.03% (7/231), capillary bronchitis 16.45% (38/231), pneumonia (mild/severe) 76.62% (177/231) (68.40% (158/231) in mild and 8.23% (19/231) in severe cases), bronchial asthma combined with pulmonary infection 3.46% (8/231).

Type 7 multilobar infiltration, pleural effusion proportion and calcitoninogen were significantly higher than type 3 and other types, and white blood cell count was lower than type 3 and other types, and the difference was statistically significant (*P* < 0.05); There was no difference in comparing with acute bronchitis, capillary bronchitis and pneumoniaamong 3 groups, see Table 2.

**Table 2:** Clinical diagnosis in hospitalized children with acute respiratory tract infection according to the Adenovirus types

Indicators	Type 3 (*n* = 115)	Type 7 (*n* = 96)	Other type (*n* = 20)	χ^2^/t	*P*
Acute bronchitis	3(2.61)	3(3.13)	1(5.00)	0.337	0.845
Capillary bronchitis	19(16.52)	15(15.63)	4(20.00)	0.231	0.891
Pneumonia (mild/severe)				2.237	0.692
Mild disease	91(79.13)	72(75.00)	14(70.00)		
Severe	14(13.33)	24(25.00)	2(10.00)		
Bronchial asthma combined with lung infection	4(3.48)	3(3.13)	1(5.00)	0.174	0.917
Combined underlying disease	9(7.83)	10(10.42)	3(15.00)	1.167	0.558
Chronic kidney disease stage 5	2(1.74)	1(1.04)	0	0.487	0.784
Retinoblastoma	1(0.87)	2(2.22)	1(5.00)	1.828	0.401
Congenital disorders of pulmonary surface active substance metabolism	2(1.74)	3(3.13)	1(5.00)	0.897	0.639
Congenital heart disease	1(0.87)	2(2.22)	1(5.00)	1.849	0.397
Spinal muscular atrophy	1(0.87)	1(1.04)	0	0.207	0.900
Length of hospitalization	11.21 ± 2.29	11.87 ± 2.32	11.37 ± 2.32	2.179	0.116
White blood cell count (10^9^ × L)	19.92 ± 2.31	13.29 ± 2.18	14.92 ± 2.32	232.678	<0.001
Calcitoninogen (ng/mL)	0.25 ± 0.09	0.56 ± 0.06	0.34 ± 0.09	615.579	<0.001
C-reactive protein (mg/L)	25.28 ± 7.91	24.98 ± 6.92	25.32 ± 6.21	0.048	0.953
Multiple lung lobe infiltrates	21(18.26)^#^	40(41.67)^*^	5(25.00)	6.349	0.042
Pleural effusion	11(9.57)^#^	21(21.88)^*^	1(5.00)	14.672	0.001
Pulmonary atelectasis	11(9.57)	5(5.21)	2(10.00)	1.531	0.465
Critically ill adenovirus pneumonia	2(1.74)	5(5.21)	2(10.00)	3.861	0.145
Admission to PICU	4(3.48)	9(9.38)	1(5.00)	0.588	0.745
Invasive mechanical ventilation	3(2.61)	8(8.33)	1(5.00)	0.723	0.697
Death	1(0.87)	2(2.08)	1(5.00)	2.367	0.306

^#^*P* < 0.05 compared with type 7

^*^P < 0.05 compared with other types

## Discussion

In our study, it was found that the adenoviruses infected in hospitalized children with ARTI were mainly type 3 and type 7. Higher percentage of shortness of breath, multilobar infiltration, and pleural effusion were observed in clinical symptoms of type 7. A study noted that the main type of AdV infections among inpatients and outpatients in south China were type 7 [11]. A study by Yali Duan [12] et al. noted that a cross-sectional study of nine hospitals in different provinces of China pointed out that the predominant types in the northern local epidemic were types 3 and 7, and the predominant types in the southern region were types 2 and 3. In addition, a shift in the predominant AdV endemic type from type 3 to type 7 was monitored in Guangdong, and this change in type may have caused the AdV outbreak in the southern region in 2018 [13]. The findings of these studies are generally consistent with our results, implying that there are no significant differences in the AdV types leading to ARTI across Chinese provinces.

ARTI is a common disease in children, which can occur throughout the year and has a high morbidity and mortality rate, seriously affecting the lives of people worldwide, and AdV infection is one of the important pathogens causing ARTI in children [14]. AdV is commonly found in nature and AdV infections have been reported worldwide. Since 1953, when Rowe first isolated AdV, and 1958, when research on AdV infections began in China, previous reports indicated that 100 serotypes have been identified, divided into two genera, mammalian AdV and avian AdV, while human AdV is divided into 7 subgroups A-G, with more than 80 different serotypes, which can exist as regional, epidemic and sporadic infections with worldwide, or outbreaks epidemics [15]. Different types of human AdV have different characteristics such as histophilia, pathogenicity, and endemic areas, and are mainly transmitted through droplets, contact, and fecal-oral, etc. Patients with AdV infection and those with occult infection are the main sources of infection, which can cause pneumonia, pharyngeal-conjugate fever, tonsillitis, cystitis, encephalitis, and enteritis, etc. Most patients have mild or insignificant symptoms, but the infection can also spread to the whole body and severe pneumonia, and even death; in addition, AdV infection can leave different degrees of sequelae, and most patients have a poor prognosis, which has a serious impact on the quality of life of the children [16]. Therefore, it is necessary to analyze the typology and clinical characteristics of AdV infection in children with ARTI in order to provide a basis for clinical prevention and treatment.

As AdV recombination leads to the continuous emergence of novel types, the International AdV Organization believes that the acquisition of the three gene sequences, five-neighborhood, six-neighborhood and fibronectin, is necessary to determine the AdV type [13]. The results of the present study showed consistent results of typing of 231 cases with 3 genes, where 49.78% (115/231) of AdV type 3, 41.56% (96/231) of type 7 and 8.66% (20/231) of other types were identified, which is consistent with the results of the above study. The results of Lin [17] et al. pointed out that type 7 infections have lower white blood cell counts than type 3 infected children and higher calcitonin pro and C-reactive protein levels were high. The results of the present study showed that type 7 wheezing or shortness of breath, multilobar infiltrates, pleural effusion proportion and calcitoninogen were significantly higher than type 3 and other types, and leukocyte count was lower than type 3 and other types (*P* < 0.05), suggesting that the inflammatory response is higher in children with ARTI with AdV-positive gene type 7 who have more symptoms of wheezing or shortness of breath and a reduced leukocyte count and higher levels of calcitoninogen. The results of the present study differ from the above-mentioned studies in that the results of the present study did not find any difference in C-reactive protein levels between type 3 and type 7 infections. In addition, the comparison of critical AdV pneumonia, admission to pediatric intensive care unit (PICU), invasive mechanical ventilation and death among type 3, type 7 and other types in this study (*P* > 0.05), but type 7 critical AdV pneumonia, admission to PICU, invasive mechanical ventilation and death were slightly higher than type 3 and other types, which may be analyzed because type 7 AdV infection is more likely to progress to severe disease. The study by Xie [18] et al. noted that type 7 AdV infections are more likely to progress to severe pneumonia, with higher oxygen requirements and longer hospital stays. Similarly, Fu [19] et al. showed through in vivo, in vitro and clinical correlation analysis that type 7 AdV has a greater replication capacity compared to type 3 AdV and can promote and exacerbate cytokine responses, resulting in a more severe respiratory inflammatory response.

There are shortcomings in this study. The results of this study are retrospective and only represent the typology and clinical symptoms of AdV infection in children admitted to our hospital with ARTI. In addition, significant differences in the distribution of AdV infection subtypes between children in the north and south of China have been previously found, and subsequent multicenter studies need to take into account sampling in the north and south. Moreover, the recently discovered association of severe acute hepatitis with AdV and SARS-CoV-2 should be included in subsequent studies if possible.

## Conclusion

In summary, type 3, 7 and other types of AdV infection are common in children hospitalized with ARTI. Type 7 has a higher proportion of shortness of breath, multilobar infiltration, pleural effusion, significantly higher calcitoninogen, and lower in white blood cell count. These results indicating that type 7 is more severe and should be given clinical attention and timely and useful treatment. Therefore, clinical attention should be paid to differentiate AdV types, and timely monitoring of AdV helps clinicians to diagnose, treat and judge the prognosis of the disease.

## Data Availability

The datasets generated and analyzed during the current study are available from the corresponding author on reasonable request.

## Abbreviations

DFA: direct immunofluorescence assay
RPP: respiratory pathogen detection kit
